# Contrasted patterns of evolution of the LINE-1 retrotransposon in perissodactyls: the history of a LINE-1 extinction

**DOI:** 10.1186/s13100-018-0117-4

**Published:** 2018-03-28

**Authors:** Akash Sookdeo, Crystal M. Hepp, Stéphane Boissinot

**Affiliations:** 1 Department of Biology, New York University, New York, NY USA; 2 School of Informatics, Computing, and Cyber Systems, Northern Arizona University, Flagstaff, AZ USA; 3 New York University Abu Dhabi, Saadiyat Island, Abu Dhabi, United Arab Emirates

**Keywords:** LINE-1, Retrotransposon, Molecular evolution, Horse, Rhinoceros, Perissodactyla

## Abstract

**Background:**

LINE-1 (L1) is the dominant autonomously replicating non-LTR retrotransposon in mammals. Although our knowledge of L1 evolution across the tree of life has considerably improved in recent years, what we know of L1 evolution in mammals is biased and comes mostly from studies in primates (mostly human) and rodents (mostly mouse). It is unclear if patterns of evolution that are shared between those two groups apply to other mammalian orders. Here we performed a detailed study on the evolution of L1 in perissodactyls by making use of the complete genome of the domestic horse and of the white rhinoceros. This mammalian order offers an excellent model to study the extinction of L1 since the rhinoceros is one of the few mammalian species to have lost active L1.

**Results:**

We found that multiple L1 lineages, carrying different 5’UTRs, have been simultaneously active during the evolution of perissodactyls. We also found that L1 has continuously amplified and diversified in horse. In rhinoceros, L1 was very prolific early on. Two successful families were simultaneously active until ~20my ago but became extinct suddenly at exactly the same time.

**Conclusions:**

The general pattern of L1 evolution in perissodactyls is very similar to what was previously described in mouse and human, suggesting some commonalities in the way mammalian genomes interact with L1. We confirmed the extinction of L1 in rhinoceros and we discuss several possible mechanisms.

**Electronic supplementary material:**

The online version of this article (10.1186/s13100-018-0117-4) contains supplementary material, which is available to authorized users.

## Background

The LINE-1 or L1 retrotransposon (Long Interspersed Nuclear Element 1) is found in virtually all vertebrate genomes but it is only in mammals that it has accumulated to extremely large numbers, accounting for the large genome size typical of this vertebrate class [1, 2]. L1 is by far the most abundant autonomous mobile element in mammalian genomes and in most species it is the only autonomously and actively replicating transposable element [2, 3]. The rate at which L1 accumulates in the genome of its host differs through time and among lineages [4–8]. Evolutionary analyses revealed that waves of intense amplification alternate with periods of low amplification [5, 9]. This pattern could be caused by a number of factors including evolution of novel L1 families that have the ability to escape host repression [5, 10, 11], competition between L1 families [4, 12, 13], and the demographic history of the host [1, 14–17]. Although L1 is active in most extant mammals, it is apparently extinct in several lineages of rodents, cetaceans, bats and Afrotheria [6, 18–21]. What has caused the complete extinction of L1 in these lineages is unknown. Competition with other types of mobile elements [22, 23] is a possible explanation. However, the stochastic loss of progenitors could also account for this loss of activity because full-length copies tend to be found in small number and at low frequency in natural populations [16, 24]. This question has not been addressed in detail, possibly because mammalian genome sequences of high quality are just becoming available for this type of study. It is however an important evolutionary question because species with no active L1 will be spared the fitness cost imposed by L1 activity [16, 25–27] but they will also be deprived from a significant source of genetic novelties [28–31].

The availability of complete genome sequences across the entire tree of life has led to a better understanding of the biology of L1 in a wide diversity of eukaryotes [6, 18, 32–41]. However, most of what we know about L1 evolution in mammals comes from the detailed analysis of two orders, primates and rodents, both belonging to the euarchontoglires super-order [5, 7, 9, 12, 13, 19–21, 23, 42–46]. In these two groups L1 generally evolves as a single lineage of family, so that only the most recently evolved family is active [4, 5, 47]. This single lineage mode of evolution is unusual, yet is a typical feature of mammalian L1, while L1 is represented by a large diversity of divergent and simultaneously active families in reptiles, amphibians and fish [10, 32, 37, 39]. In a few instances, it was shown that several lineages of families amplified simultaneously for extended periods of evolutionary time [5, 43, 48]. This seems to occur only when these lineages carry non-homologous 5′ untranslated regions (UTR) and it was proposed that families with different 5’ UTRs can coexist because they may not rely on the same host-encoded transcription factors and therefore occupy different niches in the genome of their host [5]. It was also found that a region of the first open reading frame (ORF1) evolves rapidly at the amino acid level, suggesting this region is evolving adaptively [48, 49]. This pattern of rapid evolution seems to coincide with periods of intense amplification and is consistent with an arms race model of evolution between L1 and its host. However, those “rules” of L1 evolution have been established by the analysis of a small number of taxa, mostly human and murine rodents. With the availability of numerous mammalian genomes, a comprehensive study of L1 evolution in other mammalian orders is warranted [2].

Among the lineages that could best illuminate the evolution of L1 and the cause(s) for loss of activity are the Perissodactyls, a mammalian order that includes horses and rhinoceroses, and belongs to the laurasiatheria super-order. The horse genome is known to harbor active L1 s [50] whereas L1 may be extinct in the rhinoceros genome [6]. High quality genome sequences are available for the domestic horse [51] and for the white rhinoceros allowing for a unique opportunity to investigate the process of L1 extinction. Another advantage of studying this group is that it has the best fossil record among mammals [52], so that the time of divergence between species can be established with a high level of certainty. Here we performed a detailed analysis on the evolution of L1 in the order perissodactyla. We found that L1 has diversified and persisted in the horse genome but became extinct in the rhinoceros lineages, although the last active L1 families in this genome did not show sign of decreased activity until their relatively sudden extinction. We also found that several lineages of L1 families have evolved in horse but that these lineages coexisted only when they harbored different 5’UTR, suggesting that similar mechanisms are limiting L1 diversity in perissodactyls, rodents and primates.

## Methods

### Collection and classification of full-length L1 elements

Full-length (FL) elements were collected from the *Equus caballus* 2007 (EquCab2) and *Ceratotherium simum simum* 2012 (cerSim1) genome assemblies using GPS [53]. These two genome sequences were generated by the Broad Institute, Cambridge, MA, and were downloaded from the genome.ucsc.edu website. First, GPS conducted a BLAST type-search (WU-tBLASTn) of each genome using the conserved Reverse Transcriptase (RT) domain of ORF2 as a query motif. GPS then extracted 7000 bp upstream and downstream of the RT domain yielding fragments roughly 14,000 bp in length. A second WU-tBLASTn was then performed on each fragment to identify other motifs characteristic of FL mammalian L1 inserts (3’UTR, ORF2, ORF1). In this analysis, GPS did not search for sequence identity at the promoter region since L1 has been documented to frequently undergo recruitment of novel sequences as 5’ UTRs. A file containing FL fragments and their 3000 bp upstream regions was generated for each genome. In addition, we collected all FL elements that were already annotated in these two genomes to insure we didn’t miss any L1 subfamily.

FL sequences were categorized into families based on their phylogenetic relationships along the entire length of each sequence. A family is defined as a collection of inserts that result from the amplification of a highly homogenous group of progenitors that are easily identifiable by their unique combination of characters. We used neighbor joining trees to compare the global relationships of elements as a first step in our phylogenetic analysis. Distinct clusters of elements were considered to be families and were validated by a second round of phylogenetic analysis on the 5′ promoter region to confirm the existence of distinct families that originated from the replicative activity of highly similar progenitors. FL consensus sequences were derived for each family and are available as Additional file 1 and Additional file 2. Phylogenetic analyses were performed using the neighbor joining (NJ) method based on the maximum composite likelihood parameters included in the MEGA 6.06 software package [54]. The robustness of each phylogenetic tree was assessed using the bootstrap technique with 1000 replicates. Families were named based on which genome they amplified in, a number corresponding to their 5′ promoter, followed by a Roman numeral. The smaller the Roman numeral, the younger the family is. Horse specific families are indicated as Ec, rhinoceros families as Cs, and families that amplified before the split between these two species are referred a perissodactyla families. The consensus sequences of all families is available as Additional file 1 and Additional file 2.

### Analysis of FL elements

NJ and maximum likelihood (ML) trees were calculated for each region and domain of L1. Phylogenetic trees were calculated using the MEGA 6.06 package. The RDP4.0 program (Recombination Detection Program 4.0) was used to detect events of recombination between families of L1 [55]. RDP software allows for the use of substitution and phylogeny-based methods to identify possible breakpoints. Two substitution-based methods, MaxChi and Chimaera, as well as a phylogenetic method, Bootscan, were used to analyze the dataset [56–58]. The RDP software also includes its own unique algorithm termed ‘RDP’ which is also phylogenetic-based method [59]. A window size of 50 bp was used to detect breakpoints between consensus sequences and statistically significant events of recombination were verified by comparing phylogenetic trees on each side of the putative breakpoint.

To test for evidence of selection, several methods from the http://www.datamonkey.org/ web server were used [60]. PARRIS uses a maximum likelihood approach to determine if a proportion of sites in an alignment evolves with a ω ratio dN/dS > 1 [61]. A ω ratio significantly > 1 is characteristic of positive selection whereas a ratio < 1 is characteristic of purifying selection. GABranch can detect lineage-specific variation under-going selective pressure within a dataset and requires no a priori specification of branches in a phylogeny [62]. To detect evidence of codon specific selection within an alignment, we used three methods: Single Likelihood Ancestor Counting (SLAC), a Random Effects Likelihood (REL), and Fixed Effects Likelihood (FEL) [63]. The datamonkey server allows for the automatic detection of the model that best fits the dataset. This tool was used for each individual dataset. As selection detection methods are sensitive to recombination, we performed our analyses independently for each segment of L1 flanked by a recombination breakpoint.

Tandem Repeat Finder [64] was used to detect repetitive motifs within the 5′ promoter regions of L1 inserts, which have been described in previous studies performed in mouse and other mammals [4, 32, 48, 65–70]. Previous studies on human L1 have documented positive selection in the coiled-coil (CC) domain of ORF1 [5, 49]. CC structures are formed from two or more α-helical peptide chains that contain a distinct arrangement of non-polar side chains. The program COILS was used to identify the position of the CC domain in each consensus sequence as well as the number of consecutive heptads [71].

### Age and copy number of L1 families

The age of each family was estimated by calculating the average pairwise divergence based on the 3′ end of each family of elements. Since the 3’ UTR in perissodactyla is short (~ 100 bp), a 500–600 bp region of the end of ORF2 and the 3’UTR was used to estimate family age. CpG dinucleotides and the highly mutable polypurine tracts located in the 3’UTR were removed from alignment. The average distance between copies, as well as the standard error, was calculated using the maximum likelihood parameter distance (using the MEGA 6.06 suite). Divergences were converted to time assuming a divergence of *Hippomorpha/Ceratomorpha* at 56 Myr, which is consistent with molecular and paleontological studies [52, 72, 73].

Family copy numbers were estimated using a locally installed copy of the RepeatMasker software. A custom library was constructed using our consensus sequences and used as a query to identify genomic L1 inserts in the complete genome sequences of *Ceratotherium simum* and *Equus caballus.* Since L1 elements tend to decay with time because of internal deletions [36, 37, 74–76], we provide two estimates of FL copy number. The first estimate is based on the number of 5’UTRs generated by each family. L1 transposition occurs at the site of insertion and the reverse transcription start at the 3′ end of L1 RNA, thus the presence of a 5’UTR indicates that at the time of insertion a complete element was produced. The second estimate is the number of element identified by GPS with a complete 5’UTR.

### Genome wide investigation of indels and divergence variation

Whole genome alignments were constructed using *E. caballus* chromosomes 1,2,3,5,7,9 and X as a reference genome to available *C. simum* contigs. These regions were chosen based on the higher quality of reference genome sequence available. The alignments were then analyzed using a locally run build of RepeatMasker using our consensus sequences as a custom library to identify L1 inserts located within these specific regions. Insertions, deletions, and divergence from consensus was calculated for 500 loci each, for two subfamilies of L1 that amplified and went extinct before the split of the Hippomorpha and Ceratomorpha suborders: L1_Perissodactyla4_II and L1_Perissodactyla6_I.

## Results

A total of 2084 and 4180 full-length (FL) L1 inserts were recovered using GPS or the RepeatMasker tables from the *E. caballus* and *C. ceratotherium* reference genomes, respectively. L1 elements were then aligned along the full length of the inserts and grouped based on their 5’ UTRs. Elements showing homology in their 5’UTR regions were then aligned along the full length of the insert and further categorized into families using a phylogenetic analysis of their 3′ terminus regions. A family here is defined as a collection of elements that result from the activity of a highly homogenous group of progenitors, which are classified by a unique combination of distinct characters. Neighbor joining trees of these elements were then constructed using different regions and functional domains of L1 to ensure that the homogeneity of the families extended over the entire length of the element. Using this approach, we identified 30 families and consensus sequences were derived for each of them (Table 1). Twenty of those families were horse specific (L1_Ec), four were rhinoceros specific (L1_Cs) and six amplified before the split between horse and rhinoceros (L1_Perissodactyla). A number was added to distinguish the seven different types of 5’UTR and a roman numeral distinguishes subsets of elements sharing the same 5’UTR. A file with the consensus sequences of the 5’UTRs is available as Additional file 1 and Additional file 2.

**Table 1:** Copy number, length, divergence and age of Perrisodactyla L1 families. The families are organized by promoter types (from 1 to 7)

		Divergence and age of families based on their 3′ extremity			Genomic Copy #		Genomic FL Copy #	
Family Name	Length of consensus (bp)	Average pairwise divergence	Average divergence from consensus (% ± S.E.)	Age (Myr)	Ec	Cs	Ec	Cs
L1_Ec1_I	6639	0.70 ± 0.20	0.43 ± 0.38	2.7 (1.9–3.5)	571	–	94 (57)	–
L1_Ec1_II	6644	0.80 ± 0.20	0.46 ± 0.11	3.1 (2.3–3.8)	532	–	102 (64)	–
L1_Ec1_III	6539	0.80 ± 0.20	0.43 ± 0.09	3.1 (2.3–3.8)	420	–	2 (3)	–
L1_Ec1_IV	6411	1.40 ± 0.20	0.69 ± 0.08	5.4 (4.6–6.2)	1589	–	147 (77)	–
L1_Ec1_V	6664	1.80 ± 0.20	1.08 ± 0.19	6.9 (6.2–7.7)	635	–	117 (73)	–
L1_Ec1_VI	6716	4.70 ± 0 .40	2.64 ± 0.24	18.1 (16.5–19.6)	630	–	73 (16)	–
L1_Ec1_VII	7914	3.30 ± 0.10	1.68 ± 0.08	12.7 (12.3–13.1)	1285	–	88 (15)	–
L1_Ec1_VIII	6701	3.60 ± 0.30	2.02 ± 0.19	13.8 (12.7–15.0)	459	–	37 (17)	–
L1_Ec1_IX	6787	5.80 ± 0.40	3.16 ± 0.22	22.3 (20.8–23.8)	1931	–	140 (15)	–
L1_Ec1_X	6791	11.20 ± 0.50	6.10 ± 0.33	43.1 (41.2–45.0)	3571	–	251 (53)	–
L1_Ec2_I	5924	1.00 ± 0.10	.051 ± 0.03	3.8 (3.5–4.2)	2561	–	403 (31)	–
L1_Ec2_II	5918	4.40 ± 0.30	2.47 ± 0.21	16.9 (15.8–18.1)	1350	–	166 (11)	–
L1_Ec2_III	5943	5.90 ± 0.30	3.22 ± 0.24	22.7 (21.5–23.8)	355	–	2 (0)	–
L1_Ec2_IV	5941	5.80 ± 0.30	3.12 ± .021	22.3 (21.2–23.5)	477	–	58 (5)	–
L1_Ec2_V	5950	6.00 ± 0 .30	3.20 ± 0.20	23.1 (21.9–24.2)	1423	–	137 (13)	–
L1_Ec2_VI	5963	7.70 ± 0 .30	4.13 ± 0.23	29.6 (28.5–30.8)	1839	–	196 (23)	–
L1_Ec2_VII	5986	9.60 ± 0.40	5.17 ± 0.28	36.9 (35.4–38.5)	4508	–	572 (35)	–
L1_Cs2_I	6002	5.2 ± 0.40	2.9 ± 0.25	20.0 (18.5–21.5)	–	6700	–	512 (6)
L1_Cs2_II	5686	8.7 ± 0.40	4.8 ± 0.33	33.5 (31.9–35.0)	–	2372	–	140 (4)
L1_Cs2_III	5518	9.5 ± 0.40	4.9 ± 0.24	36.5 (35.0–38.1)	–	1716	–	46 (12)
L1_Perissodactyla2_I	6127	13.90 ± 0.40	7.27 ± 0.29	53.5 (51.9–55.0)	9208	6572	476 (78)	312 (6)
L1_Cs3_I	6218	5.8 ± 0.30	3.0 ± 0.16	22.3 (21.2–23.5)	–	2755	–	234 (1)
L1_Ec4_I	6997	3.60 ± 0.20	1.95 ± 0.12	13.8 (13.1–14.6)	2622	–	141 (13)	–
L1_Ec4_II	6993	8.00 ± 0.40	4.38 ± 0.24	30.8 (29.2–32.3)	4150	–	316 (40)	–
L1_Ec4_III	7014	9.90 ± 0.40	5.34 ± 0.26	38.1 (36.5–39.6)	6549	–	251 (21)	–
L1_Perissodactyla4_I	7048	11.30 ± 0.40	5.99 ± 0.27	43.5 (41.9–45.0)	9944	38,189	661 (89)	1683 (12)
L1_Perissodactyla4_II	5896	17.2 ± 0.50	8.9 ± 0.27	66.2 (64.2–68.1)	9002	8807	94 (18)	127 (1)
L1_Perissodactyla5_I	5801	11.3 ± 0.40	6.4 ± 0.36	43.5 (41.9–45.0)	17,202	15,912	277 (33)	293 (2)
L1_Perissodactyla6_I	5828	16.40 ± 0.50	9.0 ± 0.39	63.1 (61.2–65.0)	13,988	13,692	274 (12)	308 (6)
L1_Perissodactyla7_I	6498	18.10 ± 0.60	10.7 ± 0.61	69.6 (67.3–71.9)	17,578	16,921	323 (11)	404 (10)

*Ec* horse-specific, *Cs* rhinoceros-specific; perissodacyla = families that are shared between horse and rhinoceros. Family age was estimated using a substitution rate of 0.13%/myr. The number of full-length element was estimated by counting the number of 5’UTR generated by each family and the number estimated by GPS (in parenthesis)

The length of the consensus sequences varies between 5518 to 7914 bp, depending on the length of their 5’UTRs and, to a lesser extent, the length of the intergenic region between ORF1 and ORF2. In general, older families are represented by much fewer FL elements in comparison to younger families, which is expected since L1 inserts decay over time due to the accumulation of internal deletions [37, 74]. We restricted our analysis to families that were represented by the presence of FL or near FL elements (> 10 copies) to derive accurate FL consensus sequences that contained identifiable promoters. Families that did not meet this criterion were removed from the dataset, as we were using only FL elements, that is elements with intact 5’UTR, ORF1, ORF2, and 3UTR. As a result, our dataset represents relatively high copy number families, which have inserted in the horse and rhino genomes as well as in their common ancestor. It is highly likely that ancient families exist in addition to those represented in our dataset however were missed by our approach due to their relatively low copy numbers.

### Phylogenetic analysis of L1 families

**Fig. 1:**
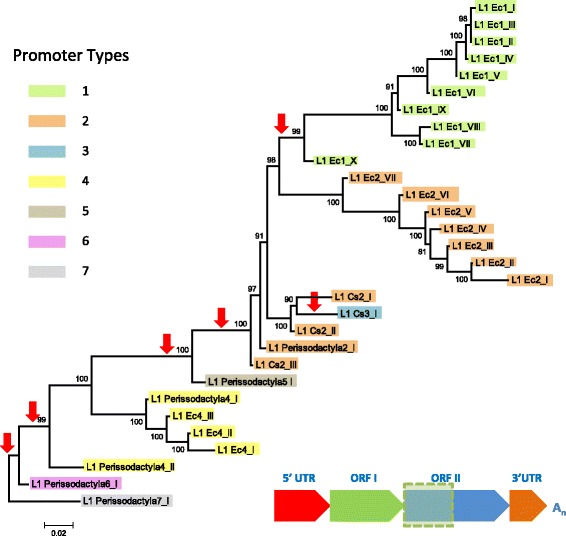
Phylogenetic tree of Perissodactyla L1 families based on the longest non-recombining region of ORF2, including the reverse transcriptase and endonuclease domains. This tree was built using the maximum-likelihood method with the HKY + G model of a non-recombining region of ORF2 as depicted in the schematic diagram of an L1 element. To test the robustness of this tree, bootstrap values for 1000 replicates were calculated and are shown at each node. Red arrows depict the acquisition of new 5’UTR sequences

**Fig. 2:**
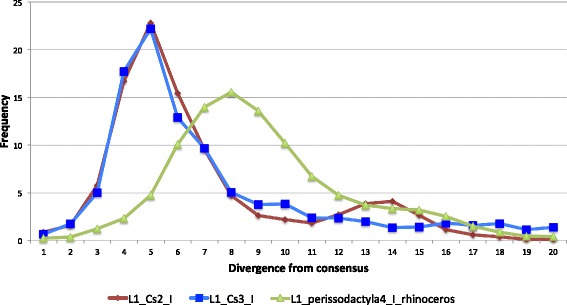
Distribution of divergence from consensus for the last three families that amplified in rhinoceros

We performed a phylogenetic analysis using the longest non-recombining region of ORF2 (2207 bp) (Fig. 1). The topology of the tree is consistent with the age of the families (Table 1) since older families are closer to the root of the tree and younger families appear more derived. The three oldest families L1_Perissodactyla7_I, L1_Perissodactyla6_I and L1_Perissodactyla4_II were active before the split between rhinoceros and horse and we estimated from their divergences that they amplified in those genomes more than 60my ago. These three families had non-homologous 5’UTRs and only L1_Perissodactyla4_II persisted, first evolving into another family shared between horse and rhinoceros (L1_Perissodactyla4_I) and eventually resulting in three horse specific families (L1_Ec4_III to I). This lineage in horse was relatively successful, generating more than 13,000 copies, until it went extinct less than 13 my ago. This lineage did not produce a clearly distinguishable rhinoceros specific family but it appears instead that the ancestral L1_Perissodactyla4_I kept amplifying in the rhinoceros genome to extremely high number (more than 38,800 copies) without undergoing noticeable evolutionary changes. This observation is surprising and suggests that some L1 families can persist over great periods of time without having to recruit novel promoters. Eventually, the ancestral promoter 4 was replaced twice, resulting in family L1_Perissodactyla5_I, which appears to be an evolutionary dead-end since it did not produce a novel lineage despite its replicative success (~ 17,000 copies), and family L1_Perissodactyla2_I whose amplification roughly coincides with the horse/rhino split and evolved into horse-specific and rhinoceros-specific lineages. The horse lineage carrying a promoter of type 2 consists of seven families, the most recent one being probably still active in the horse genome (L1_Ec2_I). This lineage alone has added ~ 13,000 L1 copies to the horse genome. The rhinoceros lineage did not diversify to the same extent, producing only three distinct families (L1_Cs2_I to III). These families were quite successful generating more than 11,000 copies but the most recent family in this lineage was active 20my ago and subsequently became extinct. In the rhinoceros, an additional acquisition of 5’UTR occurred resulting in family L1_Cs3_I. However this family appear to be an evolutionary dead-end since it became extinct at the same time as family L1_Cs2_I (Fig. 2). Finally, a novel 5’UTR was recruited in the horse genome soon after the split from rhinoceros, yielding currently amplifying lineage 1. This lineage consists of 10 distinct families that have produced more than 12,000 L1 copies.

The phylogeny and timing of amplification of L1 families reveals that multiple lineages have coexisted over extended periods of evolutionary time in both horse and rhinoceros. In horse, the lineages with the 5’UTR of type 1 (L1_Ec1_I to X) and 2 (L1_Ec2_I to VII) have coexisted for the last 40 million years of horse evolution, and until ~13my they coexisted with the ancestral lineage carrying type 4 (families L1_Ec4_I to III). The rhinoceros-specific lineage (L1_Cs2_I to III) also coexisted with the ancestral L1_Perissodactyla4_I family since before the split from horse until L1 went extinct in the rhinoceros less than 20my ago. Similarly, the genome of the ancestors of both rhinoceros and horse harbored multiple families that were simultaneously active. Thus the evolution of L1 in perissodactyls is characterized by the long-term coexistence of a small number of lineages. Different types of 5’UTRs typically characterize these lineages and we observe no or little branching within those lineages, the only possible exception being families L1_Ec1_VII and VIII, that are branching of lineage 1, yet did not persist for a long period of time. Thus it appears that the diversification of L1 in perissodactyls was driven by the acquisition of novel 5’UTRs. Our analysis indicates that novel promoters, sharing no homology with their predecessor family, were recruited a minimum of six times in the past 60 Myr (Fig. 1). These 5’UTR sequence range from 305 to 2855 bp in length and tend to be GC-rich (from 49.9 to 68.8% GC), a typical feature of L1 5’UTRs [32]. We identified two types of 5’UTRs (types 1 and 3) that contained tandem repeats, a feature shared with some mouse and rat 5’UTRs [4, 66]. The ancestral state for type 1 appears to be 3 repeats, since this is the number of repeats found in the first family with this type of 5’UTR (L1_EC1_X). One repeat was subsequently lost in families L1_Ec1_III and IV but a third copy was regained in families L1_Ec1_I and II. One family from this lineage (L1_EC1_VII) has a much longer 5’UTR than other member because of the presence of a ~ 1200 bp sequence upstream of the canonical type 1 5’UTR. This suggests that a novel site of transcription initiation was recruited upstream of an element, resulting in an elongated 5’UTR.

Although the number of L1 copies accumulated in the horse and rhinoceros genomes since their divergence is equivalent (37,000 in horse vs ~ 43,000 in rhinoceros), the dynamics of L1 amplification could not be more different in these two species. The majority of the amplification in rhinoceros (almost 29,000) result from the continuous activity of the ancestral L1_perissodactyla4_I family after the split from horse, the four rhinoceros-specific families (L1_Cs2_I to III and L1_Cs3_I) contributing an additional ~ 14,000 copies. In contrast the three lineages that have been simultaneously active in horse for the last 56 my have each contributed ~ 13,000 copies, although they diversified into 20 recognizable families and for two of them maintained activity until present. Since L1 has been extinct in rhinoceros for less than 20my, these data suggests that, until its extinction, L1 was much more prolific in the rhinoceros than in the horse genome. The last three families to amplify in rhinoceros (L1_Cs2_I, L1_Cs3_I and L1_Perissodactyla4_I) were in fact very successful (Table 1), until they went extinct. L1_Perissodactyla4_I became extinct earlier than the peak of amplification of L1_Cs2_I and L1_Cs3_I, which became extinct at the same time (Fig. 2).

### Detection of recombination among Perissodactyla L1 families

**Fig. 3:**
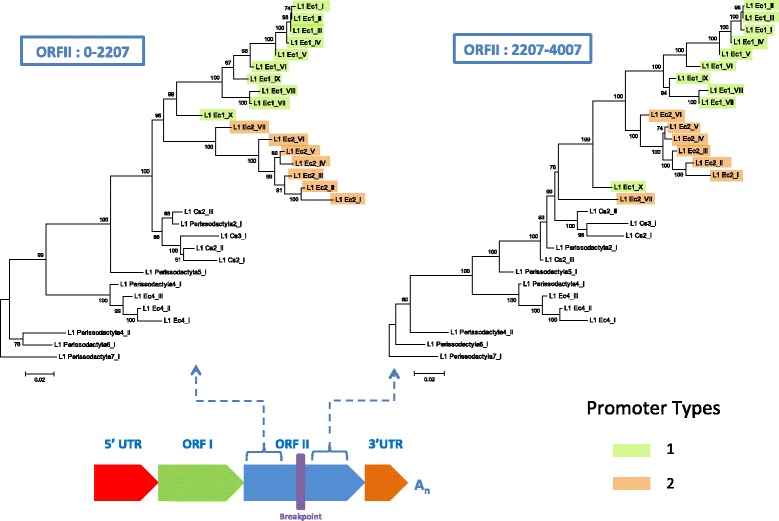
Evidence for recombination event between simultaneously active Perissodactyla L1 families. Both trees were constructed using the maximum-likelihood method with the HKY + G model of a region both upstream and downstream of the estimated breakpoint of recombination. The sequences included in each alignment of ORF2 used to construct each tree are shown within the blue boxes. Bootstrap values out of 1000 replicates are shown at each node

As observed in Fig. 1, our data suggests that L1 families have frequently recruited new promoter sequences, which allows for the possibility that they may have also exchanged genetic material in other regions of the element. To test this hypothesis, we utilized multiple recombination detection methods that were available in the RDP software package. A single event of recombination was detected. This occurred between the L1_Ec2_VII and L1_Ec1_X families (Fig. 3). The predicted breakpoint of the recombination event is located in the middle of ORF2 (position 2207). These families carry unique and non-homologous promoters and were simultaneously active between 36 and 43 Myr ago (Table 1). To test the significance of this event, two maximum likelihood phylogenetic trees were constructed using the ORF2 regions flanking the predicted recombination breakpoint (Fig. 3). The overall topology of these trees remains the same with the exception of where the L1_Ec2_VII and L1_Ec1_X branches occur. The 5′ region of ORF2 in L1_Ec2_VII more closely resembles that of the other L1_Ec2 families (shown in green). However, the 3′ region of ORF2 in L1_Ec1_X is more closely related to the Ec1 lineage (shown in blue). It should be noted that our criteria for identifying recombination events were stringent, as we only considered the recombination of large segments to be significant. It is thus possible that exchanges of shorter sequences have occurred but were not detected due to the lack of a small number of potential defining characters.

### Evolution of the ORFs

We examined the evolution of the protein encoding sequences in L1: ORF1 and ORF2. ORF2 is the most conserved region of L1. Very few amino acid substitutions between family consensus sequences are observed, supporting the idea that the endonuclease and reverse transcriptase domains are functionally indispensable. The multiple methods employed to assess the impact of selection on ORF2 indicate that this region has evolved under strong purifying selection against amino acid changes (Table 2). Our analysis looked separately at the 5′ and 3′ regions of ORF2 on each side of the predicted breakpoint because recombination can affect the outcome of tests of selection. In both regions, the PARRIS method found no evidence that any subset of amino acids was evolving under positive selection. PARRIS estimated a mean dN/dS of 0.248 and 0.267, for the 5′ and 3′ termini respectively (Table 2). GA Branch also failed to identify any branch with a dN/dS ratio being greater than one, instead the estimated values were significantly lower than 1. In addition, three alternative methods were used to identify possible specific amino acids that have evolved as a result of positive selection. FEL and REL identified two sites that may have evolved under positive selection, yet SLAC failed to identify any sites under positive selection (Table 2). It is important to note that all three methods identified a large number of sites that have evolved under negative selection; however no sites were recovered from all three methods having evolved under positive selection. Due to this discrepancy, it is likely that sites 107 and 299 of the ORF2 5′ terminus are false positives.

**Table 2:** Summary of selection detection tests

ORF	Regions	PARRIS	GA Branch		Positively selected sites	
		Mean dN/dS	Number of branches with positive selection	SLAC	FEL	REL
ORF 1	5′ terminus	0.678 ± 0.362	0	0	0	9
	Coiled coil	0.444 ± 0.319	0	0	48	0
	RRM	0.268 ± 0.258	0	0	52	0
	CTD	0.302 ± 0.398	0	0	0	0
ORF 2	5′ terminus (1–2207)	0.248 ± 0.344	0	0	107,157,299	107, 299
	3′ terminus (2207-end)	0.267 ± 0.360	0	0	0	0

We then examined the level of conservation of the domains of ORF1. Three domains have been identified: a coiled-coil (CC) domain that mediates the formation of ORFIp trimers, a RNA-recognition motif (RRM), and a C-terminal domain (CTD) [77–79]. In general, the RRM and CTD domains along with the first 50 amino acids of ORFI are very conserved across L1 families. However, the CC domain shows a high level of structural variation. We analyzed each of these four regions of ORFI independently for evidence of selection (Table 2). All of the selection detection methods indicate that ORFI has evolved under purifying selection. The PARRIS method failed to identify any subset of amino acids having evolved under positive selection with all four regions of ORFI showing a mean dN/dS well below a value of 1. GA Branch failed to identify branches with value of dN/dS > 1. While SLAC, FEL and REL identified a large number of sites under purifying selection, FEL and REL identified a total of three sites that may have evolved as a result of positive selection, however none of these sites were recovered by either of the other two methods suggesting that they were false positives.

**Fig. 4:**
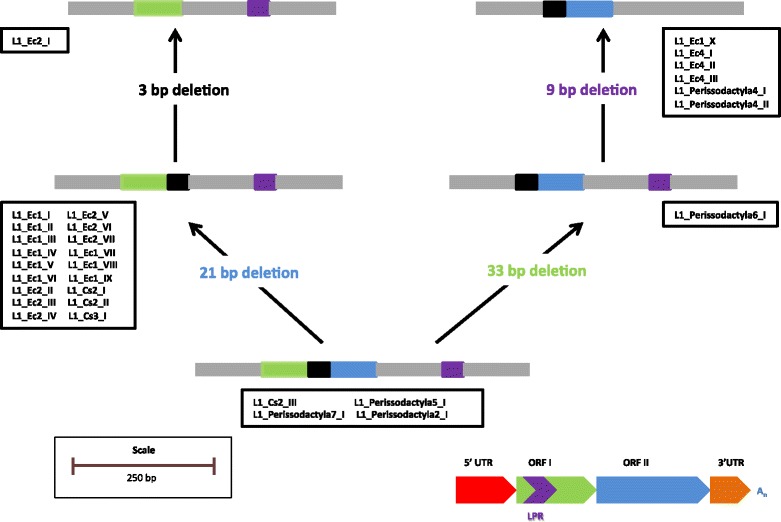
Evolution of the length polymorphic region of ORF1 in Perissodactyla. The yellow boxes correspond to a 21 bp motif, green boxes to a 33 bp motif, red boxes to a 3 bp motif and purple boxes to a 9 bp motif. Perissodactyla families that show the deletion of these motifs in ORF1 are listed within the blue text boxes. The position of the polymorphic region on the full-length elements is displayed in the bottom right of the figure

Despite this high level of conservation at the amino acid level, we observed substantial structural variation in the perissodactyla CC domain (Fig. 4). Previous studies had described this unstable region in rodents and it was called the length polymorphic region (LPR) [4]. Using our FL consensus alignment, we were able to reconstruct the history of structural changes to the LPR of ORF1 (Fig. 4). The ancestral state is found in L1_Perissodactyla7_I, L1_Perissodactyla2_I, L1_Perissodactyla5_I and L1_Cs2_III. The ancestral state, represented by these families, contain all four of the motifs that are subsequently lost in different ways in descendant families. From this ancestral state, two clades of families evolved, having lost either a 21 bp (in blue) or a 33 bp (in green) segment (Fig. 4). Of the sequences that lost the 21 bp segment (basically the two main modern lineage of horse L1 carrying 5’UTR of types 1 or 2), a further 3 bp were lost in L1_Ec2_I. Of the sequences that lost the 33 bp motif, a further 9 bp motif was deleted in L1_Ec1_X, L1_Ec4_II (and subsequent descendant families), L1_Perissodactyla4_I and L1_Perissodactyla4_II. These structural changes in the LPR resulted in changes in the length and structure of the CC domain. The CC domain plays an important role in the formation of the ORF1p trimmers [77, 79, 80] and perissodactyla L1 show extensive variation in the length and potential structure of this domain. The longest CC domain is observed in the L1_Ec1 families and contains 10 heptads with the shortest domains observed in the L1_Ec2 families that contain 5 heptads (as predicted by the program COILS).

Interestingly, we found that the region that separates the two ORFs, the IGR, differ greatly in length among families and was evolutionarily very dynamic. In ancestral families (L1_perissodactyla7_I and L1_perissodactyla6_I), the IGR was about 280 bp long. It then increased in length to about 500 bp in lineage 4, and more moderately in lineage 2, where it ranges from 476 to 328 bp. In the two oldest families with a 5’UTR of type 1, it was about 375 bp long but it shrunk to ~ 50 bp in family L1_Ec1_VIII and all its descendants.

### Analysis of genome wide neutral substitution rate

**Fig. 5:**
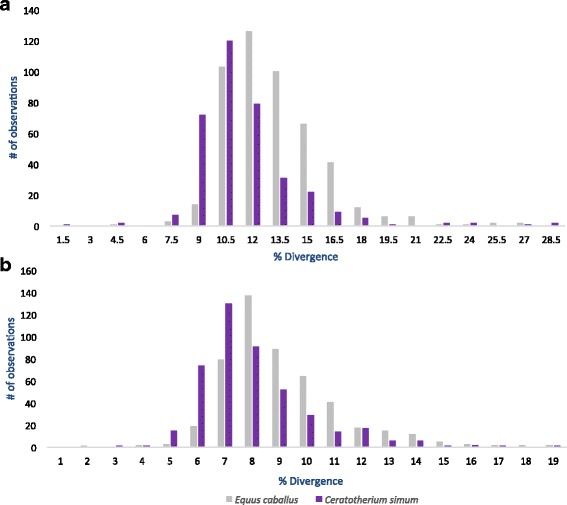
Distribution of divergence from consensus for Perissodactyla L1 families using orthologous loci. (**a**) L1_Perissodactyla4_I (**b**) L1_Perissodactyla6_I

Since the majority of L1 insertions evolved at the neutral rate, the number of differences they accumulate through time can be used to test if the rate of molecular evolution is the same across genomes. We tested this hypothesis in perissodactyls by comparing the divergence from consensus for 500 orthologous inserts in horse and rhinoceros belonging to families that amplified before the split between these species (Fig. 5). We performed this analysis on families L1_Perissodactyla4_II and L1_Perissodactyla6_I and we checked for each insert that they were orthologous in horse and rhinoceros. We observe, that in general the rhinoceros distributions are shifted toward lower values than the horse distributions, suggesting a slower rate of molecular evolution in rhinoceros. The difference between the means of the two distributions is significant (L1_Perissodactyla6_I: mean divergence from consensus in horse = 12.49%, in rhinoceros = 10.92%, *p* = 8.46*10^∧^-13; L1_Perissodactyla4_II: mean divergence in horse = 13.07%, in rhinoceros = 11.58%, *p* = 1.34*10^∧^-9) and indicate that rhinoceros evolve on average 12% slower than horses.

## Discussion

**Fig. 6:**
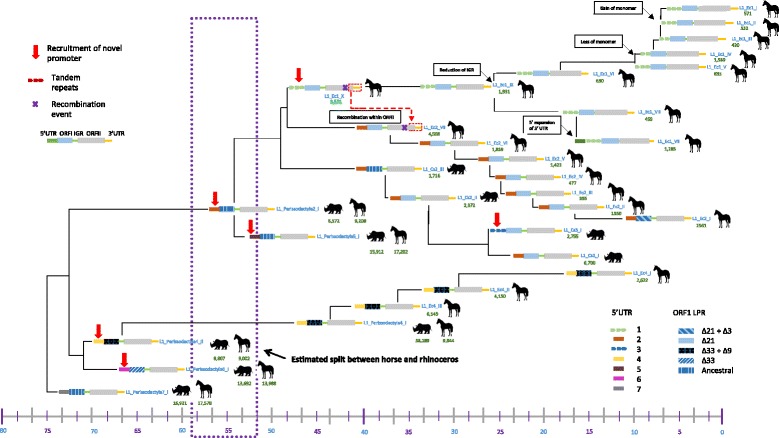
Evolutionary history of Perissodactyla L1. This composite diagram was constructed as a hand-drawn phylogenetic tree depicting the evolutionary history of Perissodactyla L1 families. Each family of L1 is represented by a schematic model of an L1 element and the blue lines that connect them show their evolutionary relationships. The numbered scale at the bottom of the diagram represents time in millions of years and the position of each L1 family reflects roughly their estimated age. Family copy numbers are shown in green text. The purple box represents the estimated split between the horse and rhinoceros between 52 and 58 Myr ago

Our analysis of the FL perissodactyla L1 dataset has yielded numerous observations that were placed into perspective on composite Fig. 6. The main result of our analysis is that L1 followed very different evolutionary trajectories in horse and rhinoceros. These two species shared three ancestral families (with different 5’UTRs) that became extinct before or at the time of the split between horse and rhinoceros (L1_perissodactyla7, 6 and 5). Two additional lineages (with 5’UTR of type 4 and 2) also emerged before the horse/rhinoceros split but persisted and amplified after the split, although their fate was very different in horse and rhinoceros. The type 4 lineage evolved into three distinct horse specific families, the last one reaching its peak of amplification ~13my ago, but only the ancestral type (L1_perissodactyla4_I) kept amplifying with great success in rhinoceros for an extended period of evolutionary time, without evolving into distinct families. The type 2 lineage evolved into seven distinct families in horse and may still be active. In rhinoceros this lineage evolved into three rhinoceros-specific families but eventually became extinct. In both species, the 5’UTR of type 2 was replaced by a novel 5’UTR, type 1 in horse, which yielded 10 families and is still active, and type 3 in rhinoceros, which produced a single family (L1_Cs3_I) that went extinct at the same time as the most recently active family of type 2 (L1_Cs2_I). The pattern of evolution of L1 in perissodactyls allowed us to investigate two questions: 1) Do the “rules” of L1 evolution inferred from the study of L1 in mouse and human apply to perissodactyls? 2) What causes the extinction of L1 in some mammals?

### The “rules” of LINE-1 evolution apply to perissodactyls

The first rule of L1 evolution is that L1 evolves mostly as a single lineage of families in mammals. Early studies in human [47] and rodents [66] had shown that L1 evolved as a single lineage: a single L1 family is active at a time and when a novel family emerges it becomes replicatively dominant and the preceding family becomes extinct. In fact, the coexistence of two families was believed to be the exception [4, 43, 44]. However, when genomic data became available it was shown that in early primate evolution three distinct L1 lineages coexisted for 30my and that the single lineage mode of evolution predominates only for the last 30my [5]. In mouse, the general trend of a single lineage applies. Although there were multiple instances of lineages branching of the main lineage, coexistence between those lineages was short lived [48]. Here we showed that three lineages coexisted for extended periods of time in horse, and two of them may still be concurrently active (lineages 1 and 2). Does this mean that the single lineage mode of evolution of L1 is wrong? Probably in its strictest formulation, but it remains that the number of L1 linages that can coexist in a mammalian genome is always small, from 1 to 3. By comparison, more than 20 L1 lineages have been simultaneously active in the lizard *Anolis carolinensis* [37] and in zebrafish [10] and the genome of the frog *Xenopus tropicalis* may contain as many as 60 distinct families [32]. Thus, it remains that there is something peculiar about the diversification of L1 in mammals. It was proposed that the single lineage reflects the nature of an arms race between mammalian L1 and its host [10, 32]. It is also possible that the demography of the host affects the rate of diversification of L1. Mammals have generally smaller effective population size than fish or reptiles and that this could limit the diversification of L1. The low diversity of L1 in fish species that have experienced bottlenecks suggests a possible impact of the host demography on the diversity of their intra-genomic parasites [36].

The second pattern that characterizes L1 evolution in mammals is the recruitment of novel 5’UTRs. First described in murine rodents [66], the recruitment of novel, non-homologous 5’UTRs seems to have occurred repeatedly in the human [5] and mouse [48] lineages. This ability to recruit novel regulatory sequence is mammalian specific since lizard, frog and fish L1 do not seem to experience a promoter turn-over [32]. It is believed that the recruitment of a novel 5’UTR provides L1 with an internal promoter the host is naïve to, which can thus escape host-encoded repressors of L1 transcription, which are co-evolving with the 5’UTR. In a series of elegant experiment, Jacobs et al. [11] confirmed this model and demonstrated an arms race between L1 and the ZNF91/93 transcriptional repressor. Our results in perissodactyls also demonstrate that the recruitment of novel 5’UTR is common in this group, with six identified recruitment events. We also showed that the first family with a new 5’UTR tends to be more successful than its successors. This is expected since the host is unprepared to repress this novel promoter. Eventually, co-evolution between the host and L1 takes place and L1 transcription is repressed more and more effectively by the host. This model is exemplified by lineage 4 in horse, where the copy number generated by this lineage started at ~ 9000 to decrease successively to ~ 6500, ~ 4100 and ~ 2600. Similarly in lineage 1 and 2, the most successful families in terms of copy numbers are the ancestral ones (L1_Ec1_X and L1_Ec2_VII).

A third rule that applies in mouse and human L1 is that coexistence between L1 lineages occur over extended periods of evolutionary times only when those lineages carry different 5’UTRs [5, 48]. This is exactly what we observe since the three lineages that coexisted for most of the evolution of perissodactyls carried non-homologous 5’UTRs (lineages 1, 2 and 4). The cause for this pattern remains somewhat unclear but we proposed that families with different 5’UTRs are not competing for the same host-encoded factors for their transcription and are therefore occupying different transcriptional niches, which allow them to coexist [5]. This hypothesis has yet to be validated experimentally.

The fourth rule is that the rate of evolution of the different regions of L1 is conserved. Not surprisingly, ORF2, which encodes endonuclease and reverse transcriptase activity [81, 82], is conserved across mammals, including perissodactyls. Of interest however is the evolution of ORF1. The C-terminal domain of ORF1 is extremely conserved across mammals [32], probably because of the presence of a non-canonical RNA recognition motif [79], and our results are consistent with this observation. In contrast, the N-terminus and in particular the coiled-coil region it contains was shown to evolve rapidly in primates and rodents, with some substantial differences. In primates, the coiled-coil domain of L1 is exhibiting a high rate of amino-acid replacement, suggestive of adaptive evolution [5, 49]. In mouse, we failed to find evidence for positive selection in the coiled-coil domain, but we found that this region was structurally unstable and had experienced a high number of insertions and deletions, resulting in changes in the overall structure of the coiled-coil [4, 48]. In perissodactyls, we did not find rapid amino acid replacements but we determined that, like in mouse, the coiled-coil domain has experienced several structural changes. We previously speculated that rapid evolution at the amino acid level and structural evolution could both have an adaptive value, either to escape a repressor of L1 transposition that interacts with ORF1 or to co-evolve with a rapidly evolving host factor necessary for transposition. Until recently, no such partner of ORF1p was known. In a recent study, the host protein TREX1 was found to repress L1 through an interaction with ORF1p [83]. This opens the door for an experimental validation of an arms race between ORF1p and a host factor, namely TREX1.

Finally, studies in rodents showed that recombination between simultaneously active L1 s generates novel families and allow for the recruitment of pre-existing motifs by emerging families for their own benefit. This recruitment of motifs was demonstrated in rats [4, 84] and we previously showed that recombinant families are very common in mouse [32]. In horse, we identified a single case of recombination between the ancestral families of the horse lineages 1 and 2. It is unclear what the functional significance of this recombination event was but our analysis suggests that recombination is not playing a substantial role in the evolution of perissodactyl’s L1 as it is in murine rodents.

### The history of a LINE-1 extinction in rhinoceros

While L1 has persisted in horse until present time, yielding novel lineages of families that amplified to great numbers, L1 appears to have gone extinct in the rhinoceros lineage and we estimated this extinction to have occurred less than 20my ago. The last substantial waves of L1 amplification in rhinoceros resulted from the activity of three families, L1_Cs2_I, L1_Cs3_I and L1_perissodactyla4_I, each carrying different types of 5’UTR (Figs. 2 and 6). The last families to have amplified in the rhinoceros genome were very successful. L1_Cs2_I, L1_Cs3_I and L1_perissodactyla4_I produced ~ 6700, ~ 2700 and ~ 38,000 copies, respectively (although ~ 10,000 L1_perissodactyla4_I copies inserted in the common ancestor of horse and rhinoceros). L1_perissodactyla4_I first went extinct before or at the time L1_Cs2_I and L1_Cs3_I amplified. Then, these two families became extinct at exactly the same time as shown on the divergence curves (Fig. 2). The possible explanations for this extinction can be classified into two categories: the explanations that are related to some intrinsic feature of L1 and the explanations involving a genome-wide effect. First, extinction can be caused by a lower rate of amplification due to some defective molecular feature that would result in a low rate of transposition, affecting in particular the insertion of FL copy (e.g. a low processivity of the reverse-transcription reaction). For instance, experiments in cells have revealed that the presence of an IGR reduces the efficiency of transposition of the extinct megabat L1, although this could not account for the extinction of L1 in this species [35]. In the case of families L1_Cs2_I and L1_Cs3_I we don’t see any evidence for a decreased rate of transposition since these families rapidly generated thousands of copies. In fact, L1_Cs2_I was replicatively more prolific than the families it succeeded. In addition, these two rhinoceros families produced a substantial fraction of FL elements (~ 10%), which is similar to other L1 families. A lower transposition rate could also be caused by the inability of L1 to evade repression by the host. More and more data are suggesting that L1 is engaged in an arms race with its host and that novel mechanisms constantly evolve to repress L1 [85], the evolution of the KRAB Zinc finger genes ZNF91/93 in primates representing a prime example of this co-evolutionary process [11]. Thus, a situation where L1 would fail to evolve a novel family, which would escape transcriptional repression by the host, could become extinct. However in the rhinoceros case, a novel 5’UTR had been recruited by the rhinoceros specific lineage (type 3) just before L1 went extinct, and the older version (type 2) was apparently very successful as demonstrated by the copy number of L1_Cs2_I.

The second category of explanations consists of mechanisms that are more global and not family-specific. This includes the evolution of novel repressors of transposition, competition with other mobile elements and the demography of the host. For instance, one can speculate that a novel mechanism of repression of L1 evolved in rhinoceros and that this repressor of transposition was so effective that it lead to the extinction of L1. The fact that L1 was so successful in rhinoceros, much more so than it was in horse, before becoming extinct would constitute a strong selective pressure on the host to evolve an effective repression of transposition. A number of defense mechanisms against transposition have evolved in mammals, and some of these mechanisms are specific to particular mammalian lineages [85]. Thus we can’t exclude that a very effective, but yet unknown, repressor of transposition has evolved in rhinoceros. Another possibility is that L1 was outcompeted by another transposable element that gained replicative supremacy or that L1 biochemical machinery was recruited by a SINE that was very efficient at hijacking L1’s reverse transcriptase. This hypothesis was proposed to account for the fact that some lineages that have lost L1 activity harbor other types of active mobile DNA in their genomes such as DNA transposons [86] or endogenous retroviruses [22, 23]. However, an examination of the Repeatmasker tables associated with the white rhinoceros genome did not reveal any type of transposon that amplified at the time of L1’s demise. Finally, the demography of a host can potentially affect the rate of fixation of insertions. When an organism has a large effective population size, selection acts more efficiently against deleterious alleles, whereas in small population, drift can cause the fixation of deleterious alleles that would be otherwise eliminated by purifying selection. Population genetics data in a number of organisms have shown that FL L1 s are more deleterious than truncated ones [14–16, 36, 87], and are thus represented at low frequency in natural populations. It is however very unlikely that selection acted so strongly against FL copies so that the rhinoceros genome contained so few FL copies that L1 went extinct. It is indeed improbable that the effective population size of the rhinoceros, a large animal with long generation time, was so large that it prevented any FL copies to reach high frequency and to persist in the population. In fact, several vertebrates with very large population size, much larger than any mammalian species, contain large numbers of active retrotransposons [3, 15]. In fact, the relative abundance of L1_Cs2_I and L1_Cs3_I FL copies contradicts the hypothesis of a significant role of demography.

## Conclusion

Here we performed a detailed analysis of L1 evolution in perissodactyls, a mammalian order belonging to the super-order Laurasiatheria which diverged from the Euarchontoglires (including rodents and primates) 92 my ago [72]. We found that the general pattern of L1 evolution in perissodactyls is very similar to what was previously described in mouse and human, suggesting some commonalities in the way mammalian genomes interact with L1. In particular, the replacement of 5’UTR and the structural instability of the ORF1 suggest that these regions are the sites of an arms race with the host and that this arms race has existed since the origin of placental mammals. We also found that L1 can experience very different evolutionary fates among taxa, from the diversification and continuous amplification observed in horse, to the eventual extinction in rhinoceros. Our analysis doesn’t provide an explanation for the extinction of L1 in rhinoceros, yet the observation that two distinct families became extinct at exactly the same time indicates that the loss of L1 activity is not caused by a family-specific mechanism.

## Additional files

Additional file 1: Full-length consensus sequences. (FAS 193 kb)

Additional file 2: Consensus sequences of the 5’ UTRs. (FASTA 29 kb)

## Abbreviations

BLAST: Basic Local Alignment Search Tool
CC: Coiled Coil
Cs: *Ceratotherium simum*
CTD: C-Terminal Domain
Ec: *Equus caballus*
FEL: Fixed Effects Likelihood
FL: Full-Length
GPS: Genome Parsing Suite
L1: Long Interspersed Nuclear Element 1
LINE-1: Long Interspersed Nuclear Element 1
LPR: Length Polymorphic Region
ML: maximum likelihood
Myr: Million of years
NJ: Neighbor Joining
ORF: Open Reading Frame
RDP: Recombination Detection Program
REL: Random Effects Likelihood
RRM: RNA Recognition Motif
SLAC: Single Likelihood Ancestor Counting
UTR: Untranslated Region
